# Protective Role of Genetic Variants in HSP90 Genes-Complex in COPD Secondary to Biomass-Burning Smoke Exposure and Non-Severe COPD Forms in Tobacco Smoking Subjects

**DOI:** 10.3390/cimb43020063

**Published:** 2021-08-03

**Authors:** Enrique Ambrocio-Ortiz, Gloria Pérez-Rubio, Alejandra Ramírez-Venegas, Rafael de Jesús Hernández-Zenteno, Armando Paredes-López, Raúl H. Sansores, María Elena Ramírez-Díaz, Filiberto Cruz-Vicente, María de Lourdes Martínez-Gómez, Ramcés Falfán-Valencia

**Affiliations:** 1 HLA Laboratory, Instituto Nacional de Enfermedades Respiratorias Ismael Cosío Villegas, Mexico City 14080, Mexico; e_ambrocio@iner.gob.mx (E.A.-O.); gperezrubio@iner.gob.mx (G.P.-R.); armandoparedes1279@gmail.com (A.P.-L.); 2 Tobacco Smoking and COPD Research Department, Instituto Nacional de Enfermedades Respiratorias Ismael Cosio Villegas, Mexico City 14080, Mexico; aleravas@hotmail.com (A.R.-V.); rafherzen@yahoo.com.mx (R.d.J.H.-Z.); 3 Programa Interinstitucional Para el Fortalecimiento de la Investigación y el Posgrado del Pacífico (Programa Delfín), Tepic 63000, Mexico; 4 Department of Respiratory Medicine, Medica Sur Clinic and Foundation, Mexico City 14080, Mexico; raulsansores@yahoo.com.mx; 5 Coordinación de Vigilancia Epidemiológica, Jurisdicción 06 Sierra, Tlacolula de Matamoros Oaxaca, Servicios de Salud de Oaxaca, Oaxaca 70400, Mexico; drmariel2504@hotmail.com; 6 Internal Medicine Department, Hospital Civil Aurelio Valdivieso, Servicios de Salud de Oaxaca, Oaxaca 68050, Mexico; filitv6cv@hotmail.com; 7 Hospital Regional de Alta Especialidad de Oaxaca, Oaxaca 71256, Mexico; ane-margo68@hotmail.com

**Keywords:** *HSP90*, SNPs, COPD, biomass-burning, smoking

## Abstract

Background: Chronic Obstructive Pulmonary Disease (COPD) is an inflammatory disease characterized by airflow obstruction, commonly present in smokers and subjects exposed to noxious particles product of biomass-burning smoke (BBS). Several association studies have identified single-nucleotide polymorphisms (SNP) in coding genes related to the heat shock proteins family-genes that codify the heat shock proteins (Hsp). Hsp accomplishes critical roles in regulating immune response, antigen-processing, eliminating protein aggregates and co-activating receptors. The presence of SNPs in these genes can lead to alterations in immune responses. We aimed to evaluate the association of SNPs in the HSP90 gene complex and COPD. Methods: We enrolled 1549 participants, divided into two comparison groups; 919 tobacco-smoking subjects (cases COPD-TS n = 294 and, controls SWOC n = 625) and 630 chronic exposed to BBS (cases COPD-BBS n = 186 and controls BBES n = 444). We genotyped 2 SNPs: the rs13296 in *HSP90AB1* and rs2070908 in *HSP90B1*. Results: Through the dominant model (GC + CC), the rs2070908 is associated with decreased risk (*p* < 0.01, OR = 0.6) to suffer COPD among chronic exposed BBS subjects. We found an association between rs13296 GG genotype and lower risk (*p* = 0.01, OR = 0.22) to suffer severe COPD-TS forms in the severity analysis. Conclusions: single-nucleotide variants in the *HSP90AB1* and *HSP90B1* genes are associated with decreased COPD risk in subjects exposed to BBS and the most severe forms of COPD in tobacco-smoking subjects.

## 1. Introduction

Chronic Obstructive Pulmonary Disease (COPD) is a complex and multifactorial treatable disease characterized by a respiratory obstruction resulting from chronic exposure and inhalation of noxious particles produced by cigarette and/or biomass burning smoke [1]. Other characteristics are chronic inflammation, sputum production, lung remodeling, emphysema and/or chronic bronchitis [2,3].

Several immunological and lung cells characterize the inflammatory process, like macrophages, neutrophils and T-cells. Along with these cells, increased levels of TNF-α, IL-1, IL-2, IL-8 and CRP are detected [4,5]. It is worth mentioning that these cells and markers have shown specific patterns depending on the exposure risk factor. COPD secondary to tobacco smoking (COPD-TS) has a T CD4 phenotype, while COPD secondary to chronic exposure to biomass burning smoke (COPD-BBS) presents a T CD8 phenotype [3,6,7,8].

Several studies have proposed a genetic component implicated in the suffering of the illness, especially polymorphism in genes related to inflammation, lung remodeling and oxidant mechanisms. Genetic polymorphisms can lead to dysregulation in the expression of several molecules that could trigger hyper inflammation or deregulation in the mechanism, unleashing severe forms of the disease [9,10,11].

Heat shock proteins (HSP) have been associated with lung cancer, fibrosis, asthma and COPD [12,13,14,15]. HSP90 family is composed of the high-conserved proteins HSP90α, HSP90β, GRP94 and TRAP1. These proteins are located in different cell compartments, accomplishing functions related to protein folding, stabilizing receptors and forming complexes to activate inflammatory processes. HSP90α, HSP90β and GRP94 overexpression have been associated with cancer, fibrosis and other interstitial lung diseases. [16,17,18] The presence of single nucleotide polymorphism (SNP) could affect the expression and protein concentration, conditioning a higher risk to suffer lung diseases like COPD.

This article describes the association between rs13296 (*HSP90AB1*) and rs2070908 (*HSP90B1*) and the risk of suffering COPD-TS or COPD-BBS in a Mexican mestizo population.

## 2. Materials and Methods

### 2.1. Population Included

We enrolled a total of 1549 subjects. Full participants were divided into two comparison groups based on environmental risk factors associated with COPD: the smokers’ comparison group included smokers without COPD (SWOC = 625) and patients with COPD secondary to tobacco smoking (COPD-TS = 294). The second comparison included subjects with chronic exposure to biomass-burning smoke without COPD (BBES = 444) and subjects with COPD secondary to biomass-burning smoke (COPD-BBS = 186). We only included Mexican mestizos, ≥40 years old, indistinct sex, without clinical evidence of another lung disease. The inclusion criteria considered for the group of smokers were consuming 10 cigarettes by day in at least 10 years and having no history of exposure to biomass-burning smoke. We select subjects with exposure to BBS at least 100 h/year and never smokers in the BBS group. Exclusion criteria included: autoimmune and other inflammatory diseases, DNA samples with low concentration and/or quality.

All patients filled out a hereditary and pathology survey. Questions about smoking and BBS chronic exposure were included. Before taking a blood sample, participants signed informed consent. All included patients were stable, without supplementary oxygen at the enrollment time, without a history of exacerbations in the previous three months and without antibiotics and systemic corticosteroid treatment in the last 3 months.

Smokers were recruited from clinical service 5, the smoking cessation groups, medical consultation from the Tobacco Smoking and COPD Research Department (DITABE) and the COPD clinic. The clinical services and research areas mentioned are part of Instituto Nacional de Enfermedades Respiratorias Ismael Cosio Villegas.

BBS exposed participants were recruited from rural towns in Oaxaca highlands and suburban areas in Tlalpan. They are part of the National Program for equality between women and men with COPD timely diagnostic campaign [19]. In addition, the spirometry was done considering the altitude in each place by calibrating the spirometer.

The Strengthening the Reporting of Genetic Association Studies (STREGA) guidelines were followed to design this genetic association study [20].

This study was reviewed and approved by Ethics in Research Committee of the INER in Mexico City (protocol numbers B10-12 and B17-14).

### 2.2. Blood Samples and DNA Extraction

Peripheral blood samples were drawn by puncture of the forearm. Samples were centrifuged at 4500 revolutions per minute (RPM) for 8 min to separate peripheral mononuclear blood cells (PMBC) in a layer. DNA was extracted from PMBC using the commercial BDtract DNA isolation kit (Maxim Biotech, San Francisco, CA, USA) and then rehydrated in TE buffer (Ambion, Waltham MA, USA). All samples were quantified by UV visible light spectrophotometry using a Nanodrop 2000 device (Thermo Scientific, Wilmington, DE, USA) and stored at −80 °C until further processing.

### 2.3. SNP Selection and Genotyping

The SNPs included in this study were chosen from previous reports with other respiratory diseases or COPD studies and minor allele frequency (MAF) > 10% reported in the 1000 genomes and/or Hapmap project for the population with Mexican Ancestry in Los Angeles.

SNPs were genotyped using specific sequence TaqMan probes (Applied Biosystems, CA. USA) and the allelic discrimination was carried out using 7300 Real-time PCR system (Applied Biosystems, Foster City CA, USA). Genotype assignment and data interpretation were conducted using the Sequence Detection Software (SDS v. 1.4, Applied Biosystems).

The amplification reaction was prepared in MicroAmp^®^ Optical 96-well Reaction Plates (Applied Biosystems; Woolston, UK); each well contained, 3 µL of adjusted DNA from one subject and 5 µL of the amplification mix, composed by corresponding TaqMan probe, TaqMan™ Universal PCR Master Mix (Applied Biosystems; Woolston, UK) and nuclease-free water (Maxim Biotech, San Francisco, CA, USA) to adjust the final reaction volume. Each plate included 3 non-template control (NTC) wells as contamination control and 1% of the samples were duplicated as allele assignment control.

### 2.4. Statistical Analysis

The population and pulmonary function data, plasma determinations and correlations were analyzed with SPSS v. 25. The Hardy-Weinberg equilibrium (HWE) was calculated with Finetti v.3.0.8 software. Genotype analysis was carried out with Pearson’s chi-squared and Fisher’s exact tests, using Epi Info v. 7.1 [21] and Epidat v. 3.1 software [22], applying dominant, recessive and over dominant models. A logistic model for data correction by covariables was carried out by Plink v. 1.07. Correlation matrix and binary logistic regressions were carried by R studio v. 3.6.

## 3. Results

### 3.1. Population Description

In the smokers’ comparison, we include 294 COPD-TS and 625 SWOC. We found an increased percentage of smoking women but an increased percentage of men diagnosed with COPD, COPD-TS present less BMI than controls. In the smoking story, we found fewer smoking years in the SWOC group, cigarettes per day and, in consequence, lower TI than in cases. We decided to include these variables in logistic regression for the smokers’ group because of significant differences in age, sex and smoking story (*p* < 0.05). In biomass smoke-exposed comparison were included 186 COPD-BBS and 444 BBES. When both groups were compared, the control group was younger than the cases, presenting higher BMI and less BSI. We did not find differences in sex distribution, but almost all participants in this comparison were women (>90%). For this comparison, we included age and BSI for the logistic regression model (Table 1).

As was expected, differences in both comparisons were found in lung function data (post-bronchodilator). Interestingly, FEV1/FVC ratio was slightly higher in the BBES group compared with the SWOC group. We compared controls’ lung function (SWOC vs. BBES) and found significant differences in FEV_1_ (*p* < 0.01), FVC (*p* < 0.01) and FEV_1_/FVC (*p* < 0.01), presenting higher lung function values BBES group.

### 3.2. Hardy-Weinberg Equilibrium and Allele Frequencies

Molecular data and populational frequency are shown in Table 2. HWE was calculated in each control group (SWOC and BBES), respectively (Table 3). For both SNPs in both groups, HWE was accomplished (Figure S1). Allele frequencies were compared for each group; no differences were found in any comparison group, but we found that in biomass smoke-exposed comparison, the minor allele for rs2070908 was C (COPD= 43.6% and BBES = 49.7%). In the case of smokers’ comparison allele G for rs2070908 is reported as the minor allele (COPD-TS = 40% and SWOC = 42.6).

### 3.3. Genotypes and Model Analysis

The co-dominant model compared genotype frequencies in order to observe the distribution (Table 4). In the smokers’ group for rs13296, we found a higher percentage of AA genotypes in cases and controls followed by heterozygous genotype. GG genotype was present in less than 10% in both cases and control for this SNP. For rs2070908, the predominant genotype was heterozygous CG in both groups (>45%), for common homozygous allele (CC) were found in >30% of samples in both groups and homozygous for minor allele were presented in <20% in both groups. No differences in the frequencies between groups were found in smokers (Table 4).

In biomass smoke-exposed comparison for rs13296, we observe a higher distribution for heterozygous genotype (>45%) in cases and controls; AA genotype was the second most common and GG with a maximum of 12% in cases. We find a different distribution of genotypes for rs2070908, with GC genotype being the most common in both groups, GG being the second most common with frequency and CC being the minor allele homozygous. When frequencies were compared, bordering differences were found for heterozygous (GC, *p* = 0.049, IC = 0.4–0.9) associated with lower risk (OR = 0.5) (Table 5).

In addition, we applied the dominant, recessive and over-dominant comparison models to describe any possible effect in the genotype distribution for the SNPs. In the smokers’ comparison, no differences for any SNPs were observed (Table 6).

In biomass smoke exposure evaluation, we found a difference in the rs2070908 for the dominant model (GC + CC, *p* < 0.01, IC = 0.4–0.9) associated with a lower risk to suffer COPD (OR = 0.6). No other association was obtained for any SNP (Table 7).

### 3.4. Allele and Genotype Distribution in Control Groups

As we observe different distribution for rs2070908 in both control groups, we decided to compare allele and genotype frequencies of this SNP in 4 different populations: Mexican ancestry from Los Angeles (MXL), Colombian in Medellin (CLM), Peruvian in Lima (PEL) and Puerto Rican in Puerto Rico (PUR). We found significant differences between BBES group SWOC, MXL, CLM and PUR (*p* < 0.05), reaching higher frequency in our BBES population. Interestingly, BBES G/rs2070908 frequency was similar with PEL population (BBES = 50.3% vs. PUL = 58.2) (Table S1).

### 3.5. Severity Analysis

COPD-TS and COPD-BBS subjects were stratified according to genotypes and we considered grouping GOLD 1 and GOLD 2 for the less severity group and GOLD 3 and GOLD 4 for higher severity. In the COPD-TS severity comparison, we found a significant association for genotype GG for rs13296 (*p* = 0.013, OR = 0.22) (Table 8). In COPD-BBS, we did not find a significant association with any SNP (Table S2).

### 3.6. Correlation Matrix

**Figure 1:**
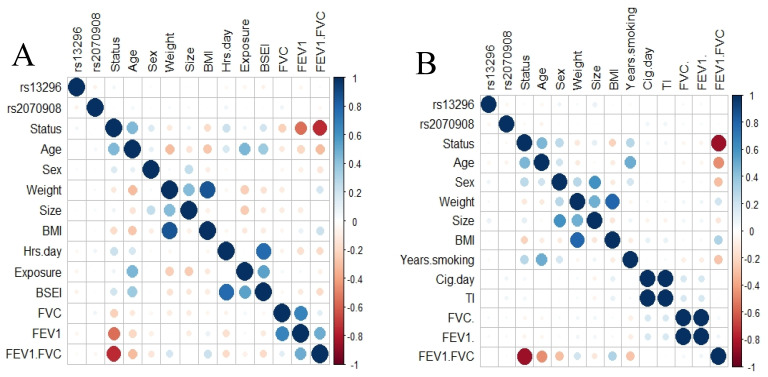
Correlation analysis. Correlation plot for smokers’ group (**A**) and exposed to biomass smoke (**B**). No correlation was found between the SNPs studied and any clinical variable.

To elucidate any correlation between SNPs and phenotype variables, we applied a correlation analysis including both SNPs. We did not found a correlation between the SNPs and any variable for COPD smokers or those exposed to BBS (Figure 1).

## 4. Discussion

This study aims to describe the possible association between COPD and single nucleotide variants in HSP90 family genes in a Mexican mestizo population. Many studies described the importance of SNPs and the susceptibility to COPD secondary to smoking, but little has been described in COPD related to BBSE. Mexico is a middle-income country, characterized by its socioeconomic heterogeneity; in 2017, 17% of the inhabitants between 12 to 65 years consumed tobacco, including those who consume more cigarettes than women (23% vs. 8.7%) [23]. On the other hand, in rural and suburban regions from Mexico, it is common to use biomass to initiate and sustain combustion for cooking, heat homes, work, or eliminate harvest debris, known as indoor pollution. In these cases, women and children are exposed commonly to these noxious particles than men. There is insufficient information about the number of families in Mexico exposed to BBS, but it is estimated that more than 30% of Mexican people are exposed.

In the current study, we included 1549 subjects, divided into two comparisons, depending on the exposure risk factor. We found sex differences, having a higher women percentage in SWOC than in COPD-TS (55.8% vs. 22.5%, respectively); this could be possible because of the social background in Mexico. In the past, women that consume tobacco were harshly judged, so they avoid consuming it. In recent years, this situation has changed; now, women are starting to consume tobacco in the same amount as men. Concerning BMI, COPD is an illness where cachexia can be experienced in severe to very severe stages. In BBS exposed comparison groups, we find statistical differences in age, BMI and BSI. We found that COPD-BBS were older than BBES (73 years vs. 60 years, respectively). In our exclusion criteria, we determine that subjects with any other respiratory disease should be excluded; commonly, women with chronic BBS exposure could suffer COPD, but at the same time, asthma or respiratory tract infections may occur when the patients get older. As in smokers’ comparison, we found diminution in BMI, a possible signal of systemic inflammation in some very severe cases [24]. Due to these differences and their importance in the illness, we included them in the logistic regression model.

This study aimed to determine the association between COPD for each risk factor and SNPs in *HSP90AB1* (rs13296) and *HSP90B* (rs2070908). rs13296 and rs2070908 were included in non-small cell lung cancer (NSCLC) and systemic lupus studies. In NSCLC, both SNPs were associated with an increased risk in a Turkish population; also, a haplotype including both SNPs was associated with increased susceptibility [25]. We only found one association study that includes rs13296 and lupus, but no significant association was found [26]. No other association studies were found related to the SNPs, even with COPD.

HSP90 is a high conserved group of genes that codifies the chaperon proteins HSP90α1 (*HSP90AA1*), HSP90α2 (*HSP90AA2*), TNF Receptor-Associated Protein 1 (*TRAP1*), HSP90β (*HSP90AB1*) and GRP94/GP96 (*HSP90B*). HSP90α and HSP90β are cytosolic proteins; GRP94/GP96 is found in the endoplasmic reticulum, while TRAP1 is localized in mitochondria [18]. HSP90α are proteins associated with the protein folding process and soluble receptor stability, as the cytosolic glucocorticoid receptor (GR). The function of TRAP1 remains unclear, but some studies have demonstrated that it accomplishes an essential role in the oxidative phosphorylation mechanism [27]. HSP90β is an 84 kD protein that can induce the production of reactive oxygen species (ROS), regulate inflammatory processes and cell cycle [28]; other studies demonstrated that this protein could be related to suffering from different lung diseases, for example, cancer, interstitial lung disease and fibrosis.

In lung cancer, up-regulation of HSP90β is associated with the worst prognosis and poor survival [29,30]; therefore, it has been proposed as a possible risk biomarker along with annexin A1 [31]; even with lung adenocarcinoma, increased levels of HSP90β have been associated to poor survival and metastasis [32,33]. In interstitial lung disease associated with autoimmune diseases, the presence of citrullination of HSP90β is proposed to trigger an immune response mediated by specific antibodies against altered epitopes and probably by autoreactive T-cells [34,35,36,37]; also, increased levels of HSP90β is associated to worse lung function in interstitial lung disease and sclerosis [38]. In fibrosis, studies related to the mechanism of fibroblast activation have demonstrated a broad relation with HSP90β. In mice with bleomycin-induced pulmonary fibrosis, HSP90β blocking demonstrated a reduction in fibrosis activation, avoiding the interaction of TGF-β receptor II- HSP90β [39]. These findings have promoted the use of therapeutics focused on inhibiting HSP90β; in mice models, the inhibitors prevent lung injuries and the production of extracellular matrix [40,41,42].

*HSP90B1* codifies the protein GRP94/GP96; it assists in the protein folding process, stability of integrins and Toll-like receptor (TLR) [43]. Studies in macrophages, dendritic, T and B-cells have demonstrated the importance of GRP94/GP96 in the innate response against infection, improving the detection and signaling related to TLR2, TLR4 and TLR9; also, in the intensification of inflammatory response mediated by cytokines, early activation of T and B-cells, improvement in the B-cell functions and, combined with cochaperone proteins participates in the folding of immunoglobulins [44,45,46,47,48,49,50,51].

Deletion of GRP94/GP96 in different cells induce a hipoinflammatory and autoimmune state via instability in TLR and other molecules; in macrophages generate TLR null cells [45], in dendritic cell reduces the immune response to sepsis [52], while in Treg-cells, the levels of FOXP3 are reduced, inducing IFN-γ and IL-17 overproduction [53].

The primary disease related to GRP94/GP96 is lung cancer; in NSLC, overexpression of *HSP90B1* is associated with poor prognosis [54]; for adenocarcinoma, increased levels of GRP94/GP96 is associated with progression [55]. These findings have made it possible to propose GRP94/GP96 as a marker for follow-up and severity in lung cancer [56,57]. Some studies have tried to use GRP94/GP96 as a therapeutic target for novel inhibitors or improvement in chemotherapy [16,58,59]. Finally, some studies have described the relation of glucocorticoids and the HSP90 proteins and showing the importance of this family in glucocorticoid resistance in COPD [60].

We did not find an association between the SNPs and COPD-TS. When the association analysis was made in BBS exposed group, we found a bordering lower-risk association with homozygous (GC) genotype and allele C for rs2070908. However, when the genetic model analysis was applied, the association values were more robust in the dominant model (GC + CC). In the rs2070908 allele frequency analysis, a significant difference in frequencies between BBS and SWOC was noted; after we compared frequencies between different Latin American populations (Mexican ancestry from Los Angeles, Colombian, Peruvian and Puerto Rican populations), we found differences in the frequency of alleles for all populations, except for Peruvian. This difference could be signaling a possible ancestral component.

rs2070908 is an SNP localized in the promotor region; genetic variants in this region could affect gene expression, primarily by altering the interaction with transcription factors. This effect could directly affect the protein quantity, downregulating some mechanisms, as proinflammatory processes. As we mentioned above, diminution in protein levels could lead to fewer inflammatory events and for that reason, we found a lower risk associated with the C allele.

The rs13296 is associated with a missense event and this can generate aberrant proteins, diminution in protein levels, or even null presence of the protein. As we previously mention, diminution levels of GRP94/GP96 could downregulate the production of inflammatory environmental; this diminution could be reflecting the association in the protective effect to develop the severe form of COPD. These hypotheses need other experimental evidence, as GRP94/GP96 and HSP90β mRNA quantification or protein levels. In addition, it could be interesting to explore other variables associated with the proposed function to HSP90, as bacterial exacerbations, or even explore the correlation of other inflammatory molecules.

In conclusion, with the evidence presented, we concluded that in the Mexican mestizo population, SNPs in genes of the HSP90 family are associated as decreased risk factors for suffering COPD secondary to biomass burning smoke and more severe forms of COPD secondary to tobacco smoking.

## 5. Conclusions

In conclusion, in the Mexican mestizo population, SNPs in genes of the HSP90 family are associated as decreased risk for suffering COPD secondary to biomass burning smoke and more severe forms of COPD secondary to tobacco smoking.

## Figures and Tables

**Table 1:** Demographic and clinical data.

	COPD-S (n = 294)	SWOC (n = 625)	*p*	COPD-BBS (n = 186)	BBES (n = 444)	*p*
Demographic						
Age (years)	66.5 (60.0–73.0)	65.5 (56–75)	<0.01	73.0 (66.5–79.0)	60.0 (54.0–69.0)	<0.01
Sex% (female/male)	78/216 (26.5/73.5)	349/276 (55.8/44.2)	<0.01	184/2 (99.0/1.0)	440/4 (99.1/0.9)	1.00
BMI	25.6 (22.5–28.2)	32.7 (28.3–37.2)	<0.01	26.1 (22.9–30.6)	28.2 (25.6–31.7)	<0.01
Exposure						
Years smoking	40.0 (30.0–49.0)	31 (30.0–32.0)	<0.01	NA	NA	
Cigarettes per day	20.0 (15.0–30.0)	15 (10.0–20.0)	0.04	NA	NA	
TI	95.0 (30.0–160.0)	40 (22.5–59.0)	<0.01	NA	NA	
BSI	NA	NA		300.0 (180.0–420.0)	210.0 (124.5–342.0)	<0.01
Lung function (post-bronchodilator)						
FEV_1_ (%)	53.0 (33.0–67.0)	78.0 (76.0–80.0)	0.04	68.0 (54.0–86.0)	104.0 (93.0–115.0)	<0.01
FVC (%)	82.0 (66.0–98.0)	78.0 (73.0–95.0)	0.08	87.0 (74.5–100.0)	99.0 (88.0–110.0)	<0.01
FEV_1_/FVC (%)	48.0 (36.0–57.9)	75.1 (72.0–83.3)	<0.01	60.0 (50.5–66.6)	83.0 (78.6–87.7)	<0.01

Data are presented in median and interquartile ranges (IR). Comparisons were evaluated through Mann–Whitney U-test. Fisher’s exact test compared sex differences. BMI: body mass index, TI: tobacco index, BSI: biomass smoke exposure index, FEV_1_: Forced expiratory volume in 1 s, FVC: Forced vital capacity.

**Table 2:** SNP’s molecular data.

SNP	Gen	Protein	Chr.	Position	Change	Effect	MAF *	MAF ⸸
rs13296	*HSP90AB1*	HSP90-β1	9	44250383	[A/G]	Missense	0.36	0.34
rs2070908	*HSP90B1*	GRP94/GP96	12	103930404	[C/G]	Promoter	0.44	0.32

Chr.: chromosome, MAF: Minor allele frequency. *: Frequency reported in 1000 genomes phase 3 for Mexican from Los Angeles. ⸸: Frequency reported in Hapmap for the American population.

**Table 3:** Hardy-Weinberg equilibrium and frequency reported.

SNP	Het. Obs.	Het. Exp.	HWE-*p*
SWOC			
rs13296	0.42	0.43	0.87
rs2070908	0.47	0.48	0.77
BBES			
rs13296	0.56	0.48	0.10
rs2070908	0.38	0.43	0.59

Het. Obs: Heterozygous observed, Het. Exp: Heterozygous expected. HWE: Hardy–Weinberg equilibrium. Fisher’s exact test calculated *p*-value and *p* < 0.05 was considered to establish not equilibrium accomplished.

**Table 4:** Genotype and allele frequencies in the tobacco smoking groups.

SNP/Genotype	COPD-S (n = 294) Freq%	SWOC (n = 625) Freq%	*p*	OR	CI (95%)
rs13296					
AA	47.3	49.4	Ref.		
AG	43.9	41.6	0.65	1.10	(0.8–1.5)
GG	8.8	9.0		1.03	(0.6–1.7)
A	69.2	70.2	0.66	0.92	(0.8–1.2)
G	30.8	29.8		1.05	(0.8–1.3)
rs2070908					
CC	35.0	33.8	Ref.		
CG	50.0	47.2	0.28	1.02	(0.8–1.4)
GG	15.0	19.0		0.75	(0.5–1.2)
C	60.0	57.4	0.3	1.12	(0.9–1.4)
G	40.0	42.6		0.89	(0.7–1.1)

Freq%: frequency in percentage, OR: odds ratio, CI: confidence interval. The χ^2^ test calculated the *p*-value and *p* < 0.05 was considered as significant.

**Table 5:** Genotype and alleles frequency in biomass smoke-exposed comparison.

SNP/Genotype	COPD-BBS (n = 186) Freq%	BBES (n = 444) Freq%	*p*	OR	CI (95%)
rs13296					
AA	37.6	42.3	Ref.		
AG	50.0	48.6	0.15	1.2	(0.8–1.7)
GG	12.4	9.0		1.5	(0.9–2.8)
A	62.6	66.7	0.19	0.8	(0.7–1.1)
G	37.4	33.3		1.2	(0.9–1.5)
rs2070908					
GG	34.8	24.3	Ref.		
GC	43.3	52.0	0.049	0.5	(0.4–0.9)
CC	21.9	23.6		0.6	(0.4–1.0)
G	56.4	50.3	0.056	1.3	(1.0–1.6)
C	43.6	49.7		0.8	(0.6–0.9)

Freq%: percentage frequency, OR: odds ratio, CI: confidence interval. The *p*-value was calculated by χ^2^ exact test and *p* < 0.05 was considered as significant.

**Table 6:** Analysis by models in the tobacco smoking comparison.

Model	Genotype	COPD-S (n = 294) Freq%	SWOC (n = 625) Freq%	*p*	OR	CI (95%)
rs13296						
Dom	AA	47.3	49.4	Ref.		
	AG + GG	52.7	50.6	0.59	1.1	(0.8–1.4)
Rec	AA + AG	91.2	91.0	Ref.		
	GG	8.8	9.0	0.95	0.9	(0.6–1.6)
Od	AA + GG	56.1	58.4	Ref.		
	AG	43.9	41.6	0.56	1.1	(0.8–1.5)
rs2070908						
Dom	CC	35.0	33.8	Ref.		
	CG + GG	65.0	66.2	0.76	0.9	(0.7–1.3)
Rec	CC + CG	85.0	81.0	Ref.		
	GG	15.0	19.0	0.15	0.7	(0.5–1.1)
Od	CC + GG	50.0	52.8	Ref.		
	CG	50.0	47.2	0.47	1.1	(0.9–1.5)

Freq%: percentage frequency, OR: odds ratio, CI: confidence interval, Dom: Dominant model, Rec: Recessive model, Od: Over dominant model. The χ^2^ test calculated the *p*-value and *p* < 0.05 was considered as significant.

**Table 7:** Analysis by models in biomass smoke-exposed groups.

Model	SNP	COPD-BBS (n = 186)	BBES (n = 444)	*p*	OR	CI (95%)
rs13296						
Dom	AA	37.6	42.3	Ref.		
	AG + GG	62.4	57.7	0.31	1.2	(0.9–1.7)
Rec	AA + AG	87.6	91.0	Ref.		
	GG	12.4	9.0	0.26	1.4	(0.8–2.5)
Od	AA + GG	50.0	51.4	Ref.		
	AG	50.0	48.6	0.82	0.9	(0.7–1.3)
rs2070908						
Dom	GG	34.8	24.3	Ref.		
	GC + CC	65.2	75.7	<0.01	0.6	(0.4–0.9)
Rec	GC + GG	78.1	76.4	Ref.		
	CC	21.9	23.6	0.71	0.9	(0.6–1.4)
Od	CC + GG	56.7	48.0	Ref.		
	CG	43.3	52.0	0.06	0.7	(0.5–0.9)

OR: odds ratio, CI: confidence interval, Dom: Dominant model, Rec: Recessive model, Od: Over dominant model. The *p*-value was calculated by χ^2^ exact test and *p* < 0.05 was considered as significant.

**Table 8:** Allele and genotype comparison for severity in COPD-TS group.

	GOLD 3 + 4 % (n = 113)	GOLD 1 + 2 % (n = 175)	*p*	OR	IC (95%)
rs13296					
AA	53.1	42.9	(Ref)		
AG	43.4	44.6	0.013	0.79	(0.48–1.28)
GG	3.5	12.6		0.22	(0.07–0.69)
A	74.8	65.1	0.014	1.59	(1.11–2.30)
G	25.2	34.9		0.63	(0.43–0.91)
rs2070908					
CC	29.2	39.4	(Ref)		
CG	55.8	46.3	0.18	1.63	(0.95–2.76)
GG	15.0	14.3		1.42	(0.68–2.99)
C	57.1	62.6	0.22	0.79	(0.57–1.12)
G	42.9	37.4		1.26	(0.89–1.77)

OR: odds ratio, CI: confidence interval. Fisher’s exact test calculated the *p*-value and *p* < 0.05 was considered as significant.

## Data Availability

The data presented in this study are available in the Supplementary Materials.

## Supplementary Materials

The following are available online at https://www.mdpi.com/article/10.3390/cimb43020063/s1, Figure S1: De Finetti plots representing HWE, Table S1: Frequency of rs2070908 in different populations in America., Table S2: Allele and genotype comparison for severity in biomass smoke-exposed group.
