# Supplementary material for: Protective Role of Genetic Variants in HSP90 Genes-Complex in COPD Secondary to Biomass-Burning Smoke Exposure and Non-Severe COPD Forms in Tobacco Smoking Subjects
Source: Curr Issues Mol Biol. 2021 Aug 3;43(2):887–99. doi: 10.3390/cimb43020063 (PMC8928934; doi:10.3390/cimb43020063)
Supplement: Supplementary file 1 [file cimb-43-00063-s001.zip › cimb-1297663-supplementary.pdf]

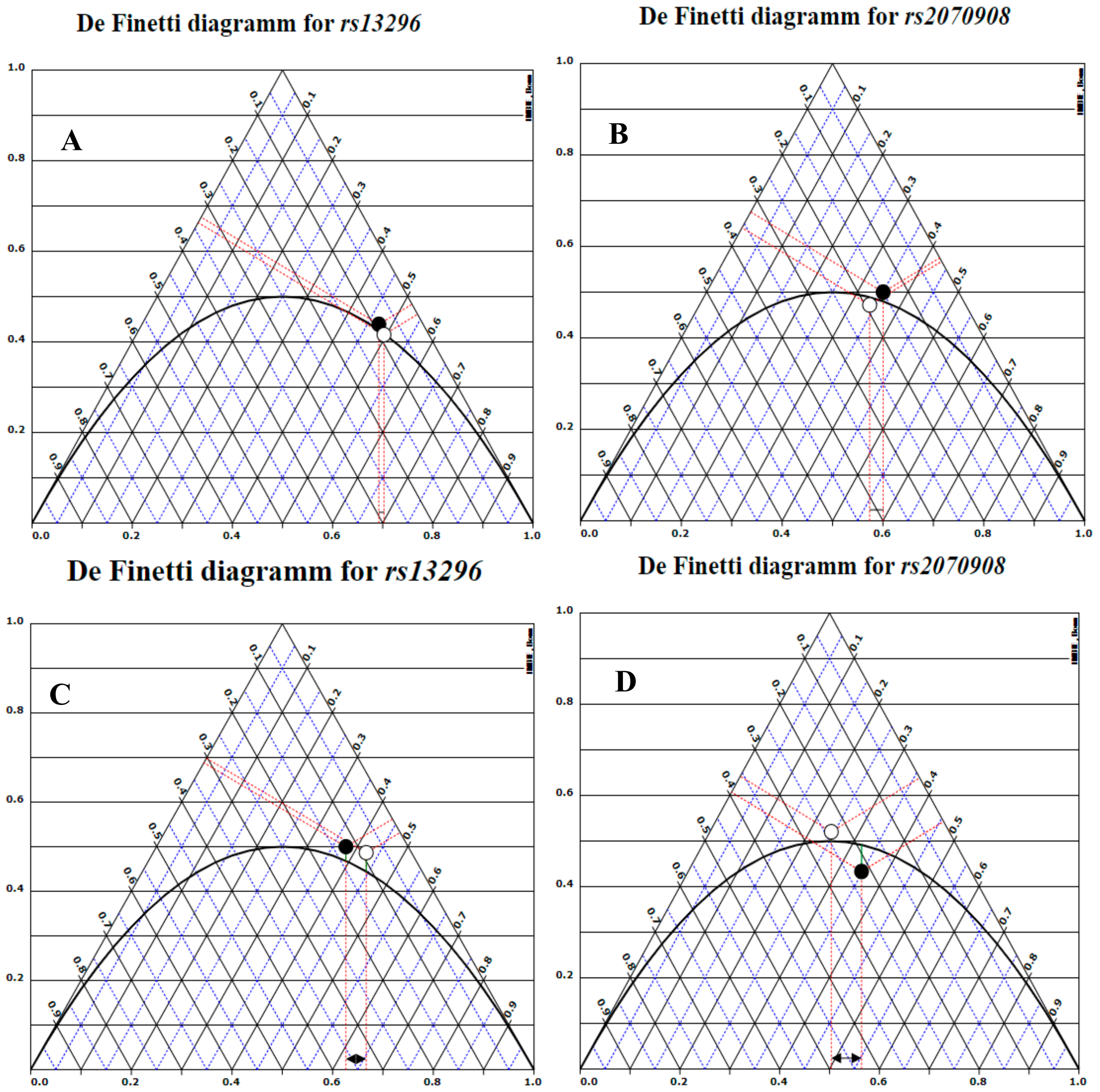

Figure S1. De Finetti plots representing HWE for smoker's comparison (A and B) and biomass exposed (C and D). White dots represent controls (SWOC or BBES) and black dots represent cases (COPD-S or COPD-BBS).

Table S1. Frequency of rs2070908 in different population in America.

	BBES (n=625, %)	SWOC (n=444, %)	MXL (n=64, %)	CLM (n=94, %)	PEL (n=85, %)	PUR (n=104, %)	p‡	p†	p*	p§	p#
**rs2070908/*HSP90B1***											
CC	23.6	33.8	39.1	38.3	17.6	31.7	0.04	0.01	<0.01	0.29	0.11
CG	52.0	47.2	48.4	41.5	48.2	54.8	0.14	0.68	0.08	0.6	0.69
GG	24.3	19.0	12.5	20.2	34.1	13.5	<0.01	0.05	0.47	0.08	0.02
C	49.7	57.4	63.3	59.0	41.8	59.1	<0.01	<0.01	0.02	0.07	0.02
G	50.3	42.6	36.7	41.0	58.2	31.7					

MXL: Mexican ancestry in Los Angeles, CLM: Colombian in Medellin, PEL: Peruvian in Lima, PUR: Puerto Rican in Puerto Rico. p value was calculated by χ^2 exact test and $p < 0.05$ was considered as significant.

‡BBES *vs.* SWOC

† BBES *vs.* MXL

* BBES *vs.* CLM

§ BBES *vs.* PEL

BBES *vs.* PUR

Table S2. Allele and genotype comparison for severity in biomass smoke exposed group

	GOLD 3+4 (n=23)	GOLD 1+2 (n=122)	p	OR	IC (95%)
**rs13296**					
AA	34.8	37.7		(Ref)	
AG	47.8	53.3	0.48	0.88	(0.32-2.41)
GG	17.4	9.0		2.09	(0.53-8.22)
A	58.7	64.3	0.57	0.78	(0.41-1.49)
G	41.3	35.7		1.27	(0.66-2.41)
**rs2070908**					
GG	17.4	28.7		(Ref)	
GC	47.8	47.5	0.18	1.66	(0.49-5.61)
CC	34.8	23.8		2.41	(0.66-8.83)
G	41.3	52.5	0.22	0.64	(0.33-1.21)
C	58.7	47.5		1.57	(0.86-2.97)

OR: odds ratio, CI: confidence interval. p value was calculated by Fisher's exact test and $p < 0.05$ was considered as significant.
